# The Recent Recombinant Evolution of a Major Crop Pathogen, *Potato virus Y*

**DOI:** 10.1371/journal.pone.0050631

**Published:** 2012-11-30

**Authors:** Johan Christiaan Visser, Dirk Uwe Bellstedt, Michael David Pirie

**Affiliations:** Department of Biochemistry, The University of Stellenbosch, Stellenbosch, South Africa; Institute of Infectious Disease and Molecular Medicine, South Africa

## Abstract

*Potato virus Y* (PVY) is a major agricultural disease that reduces crop yields worldwide. Different strains of PVY are associated with differing degrees of pathogenicity, of which the most common and economically important are known to be recombinant. We need to know the evolutionary origins of pathogens to prevent further escalations of diseases, but putatively reticulate genealogies are challenging to reconstruct with standard phylogenetic approaches. Currently available phylogenetic hypotheses for PVY are either limited to non-recombinant strains, represent only parts of the genome, and/or incorrectly assume a strictly bifurcating phylogenetic tree. Despite attempts to date potyviruses in general, no attempt has been made to date the origins of pathogenic PVY. We test whether diversification of the major strains of PVY and recombination between them occurred within the time frame of the domestication and modern cultivation of potatoes. In so doing, we demonstrate a novel extension of a phylogenetic approach for reconstructing reticulate evolutionary scenarios. We infer a well resolved phylogeny of 44 whole genome sequences of PVY viruses, representative of all known strains, using recombination detection and phylogenetic inference techniques. Using Bayesian molecular dating we show that the parental strains of PVY diverged around the time potatoes were first introduced to Europe, that recombination between them only occurred in the last century, and that the multiple recombination events that led to highly pathogenic PVY^NTN^ occurred within the last 50 years. Disease causing agents are often transported across the globe by humans, with disastrous effects for us, our livestock and crops. Our analytical approach is particularly pertinent for the often small recombinant genomes involved (e.g. HIV/influenza A). In the case of PVY, increased transport of diseased material is likely to blame for uniting the parents of recombinant pathogenic strains: this process needs to be minimised to prevent further such occurrences.

## Introduction

*Potato virus Y* (PVY) afflicts potato producers worldwide [1]–[3], causing loss of yield ranging from 10% to complete crop failure. The extent of yield reduction depends on a range of factors (including the viral load, the time of infection, temperature during growth and tuber storage and the cultivar of potato that is infected [4], [5]), but the strain of PVY involved is particularly important: some are considerably more pathogenic than others [5], [6].

Whilst potatoes have been in cultivation outside the New World since the mid 16^th^ Century, PVY was first discovered and has developed into a major crop disease only within the last 80 years. All PVY infections reduce yield, but under warmer growing conditions (such as in the potato growing regions of southern Europe and South Africa) the most detrimental strains can entirely compromise the economic viability of a crop by inducing Potato Tuber Necrotic Ringspot Disease (PTNRD). In this respect, the earliest known strains were relatively innocuous, with symptoms largely restricted to mosaic patterns or stipple streaks on leaves (PVY^C^ [7]; PVY^O^ [8]), and/or venal leaf necrosis and only rarely PTNRD (PVY^N^ [9], [10]).

More recently, genetic recombinants between PVY^O^ and PVY^N^ that induce PTNRD much more frequently have been identified. Recombination is prevalent in viruses [11]–[15] and its impact on the virulence of disease may be considerable. Foremost amongst the recently identified strains are PVY^NTN^ (N-tuber necrotic) [16] and PVY^N-W^ (N-Wilga) [17], described in 1984 and 1991 respectively [18]. Both PVY^NTN^ and PVY^N-W^ have spread rapidly, causing severe reductions in yields worldwide [19], [20]. In order to both limit the impact of existing strains on their hosts and, if possible, avoid creating the conditions that drive further escalation of pathogenicity, we need to understand the circumstances under which pathogenic recombinant virus strains such as these evolve.

However, evolutionary scenarios involving recombination are challenging to reconstruct. Individual ‘gene trees’ (phylogenies of non-recombinant regions of genomes) deviate from one another and from the underlying ‘species tree’ (representing the historical sequence of speciation events) due to differing underlying processes that are notoriously difficult to discern. Besides the various potential sources of analytical error (such as incorrect assessment of homology; model misspecification etc.), these include biologically meaningful processes such as reticulation (recombination between the branches of the species tree; i.e. between different species) and coalescent stochasticity (resulting from recombination within those branches, i.e. between individuals of the same species). It is important to distinguish reticulation from coalescent stochasticity in order to correctly infer species trees under the current methods that assume exclusively the latter process (e.g. [21]). The problem is compounded in viruses because despite generally high evolutionary rates [22] that might favour the availability of the necessary informative sequence variation, their diminutive genomes (e.g. PVY, 9.7 kb in length; HIV, 9.8 kb [11]; influenza A, 16.6 kb and polio, 7.4 kb [22]) represent a limited total source of data. Currently available phylogenetic hypotheses for PVY are either restricted to non-recombinant strains [23], [24] (i.e. excluding the most pertinent pathogenic ones), represent only parts of the PVY genome [25], [26], and/or incorrectly assume a strictly bifurcating phylogenetic tree [25], [27]–[29]. Despite attempts to date potyviruses in general [30], no attempt has been made to date the origins of pathogenic PVY.

**Figure 1:**
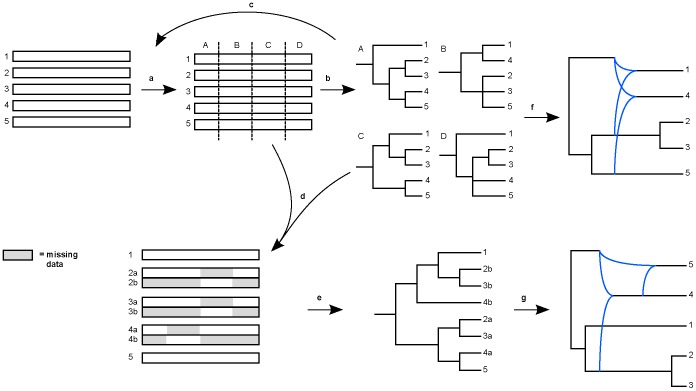
Summary of the analytical approach. The aligned sequence matrix was analysed with recombination detection software (a); the resulting breakpoints were tested with standard phylogenetic analyses of the putatively non-recombinant genome regions (b), resulting in a sequence of ‘gene trees’ (labelled A–D), and the process repeated (c), until all topological differences between gene trees could be explained by specific recombination events and vice versa. A supermatrix was then constructed following the taxon duplication approach (d; see Fig. 2); and analysed under standard phylogenetic and (relaxed) clock models (e). Phylogenetic networks were summarised from both the separate gene trees (f) and multi-labelled ‘genome tree’ (g).

In this study we reconstruct and date the phylogeny of PVY by means of a phylogenetic approach to analysing DNA sequence data in the presence of reticulation [31], [32] that we extend to address multiple recombination events between whole genomes. Unlike existing approaches, ours neither assumes a bifurcating species tree nor assumes prior knowledge of processes underlying deviations between individual gene trees. We use the resulting robust, time calibrated phylogeny to place patterns of divergence and recombination in PVY in the historical context of human cultivation of potatoes. In particular, we test whether diversification of the major strains of PVY and recombination between them occurred within the time frame of potato domestication and/or modern cultivation.

## Materials and Methods

### Sampling

We sampled PVY isolates from Africa, Asia, Europe, and both North and South America, covering all known recombinant strains for which whole genome sequences were available (Table S1). Fifteen new genome sequences were generated following direct amplification RT-PCR protocols described in [33]–[35], and 29 further sequences [24], [26], [28], [36]–[46] were obtained from GenBank. Outgroups were Pepper Mottle Virus (PMV) and two isolates of Sunflower Chlorotic Mottle Virus (SCMV); the latter more closely related to PVY than those included in previous analyses (cf. [24]). Sequences were aligned using BioEdit 7.0.5.2 [47]. Short regions of uncertain homology between outgroup and ingroup sequences were treated as insertions and excluded from analyses (Dataset S1).

**Figure 2:**
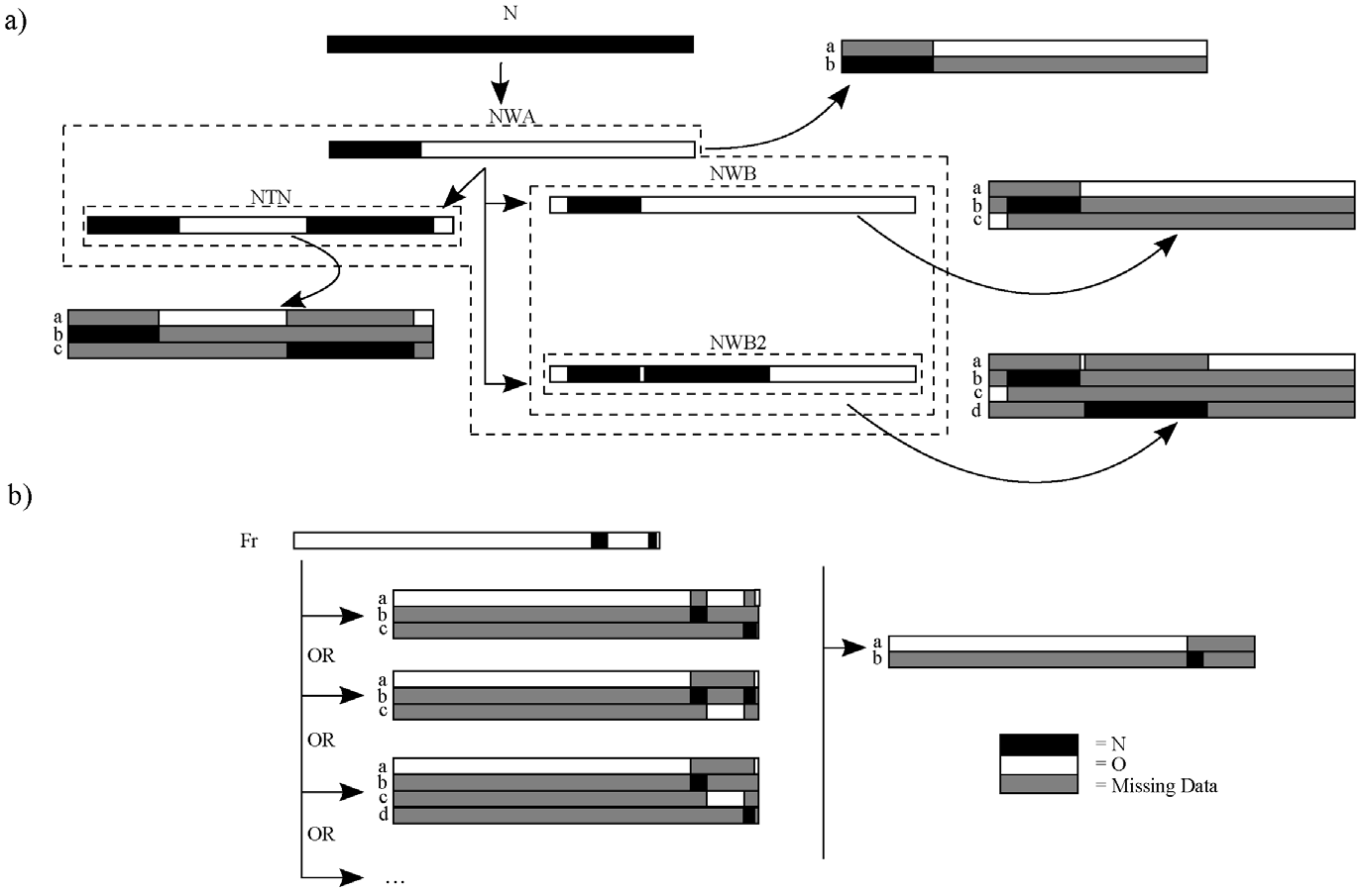
The taxon duplication approach and multiple recombinants. Recombinant genomes encode a mosaic of differing phylogenetic relationships. Depending on the pattern and sequence of recombination events, separate regions of a given genome may share a common history of inheritance whilst those immediately adjacent are more distantly related. Under the ‘taxon duplication’ approach, these distinct ‘phylogenetic signals’ are segregated into separate taxa in the data matrix. Precisely which genome regions should be combined and which should be analysed independently can be inferred from the logical sequence of recombination events. a) In the case of PVY^NW^ and PVY^NTN^, single, double and triple recombinants are apparent from shared derived recombination patterns (indicated here by black and white bars) and confirmed by exclusive ancestry (monophyly; indicated here by dotted boxes) of the pertinent genome regions. These are treated as two, three and four taxa respectively, as indicated, with the rest of the alignment re-coded as missing data. b) In the case of isolate Fr, lower recombinants are not known and phylogenetic signal is not sufficiently strong to discern congruence from conflict across the genome, thus the data could logically be combined in a number of different ways. In this case, the shorter of the non-contiguous regions are excluded from further analyses.

### Recombination Detection and Matrix Construction

Our analytical approach is illustrated in Fig. 1. We used multiple recombination detection methods as implemented in RDP3 [48] and SimPlot [12] to identify breakpoints followed by testing those breakpoints using phylogenetic analyses under parsimony and ML (as below) of non-recombinant regions to confirm the changing phylogenetic signal observed when progressing from the 5′ to the 3′ end of the linear PVY genome. Using RDP3, five methods were applied: RDP [48], [49], GENECONV [50], [51], MaxChi [52], [53], BootScan [54], [55] and SiScan [56]. Sequences were treated as linear. The threshold P-Value was set at 0.05, using Bonferroni correction. Following the RDP3 manual this should give few false positives but will still allow detection of most recombination events. The SEQGEN parametric simulations and phylogenetic evidence options were selected. For method-specific settings we followed the RDP3 manual. The results of the subsequent phylogenetic analyses of putatively non-recombinant regions were assessed for topological conflict subject to bootstrap support (BS) ≥70% under both parsimony and ML. The process was then repeated with breakpoints that did correspond to such conflict tentatively assumed to be correct until all topological differences between the ‘gene trees’ could be explained by specific recombination events and vice versa. The phylogenetic analyses represent a conservative test of the (not necessarily unanimous) results of the recombination detection methods. It will tend to reject recombination both when it has been incorrectly inferred and where it is real but involves little sequence variation (generally corresponding to very short regions and/or very recent events). We regard the latter as effectively impossible to address using phylogenetic approaches and assume that it will be of low impact on the subsequent analyses.

**Figure 3:**
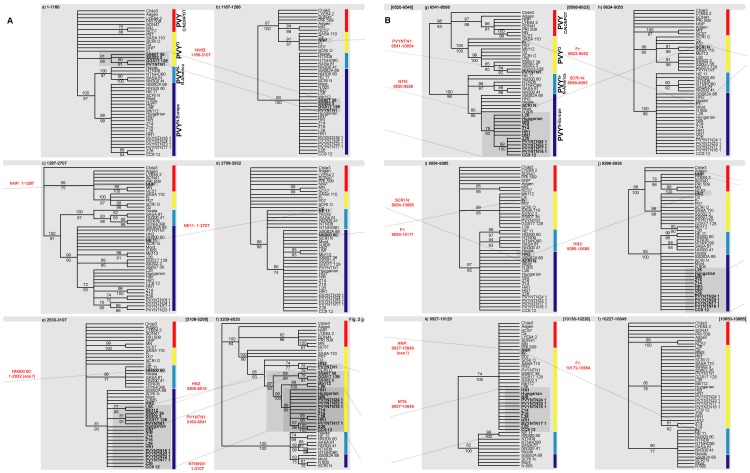
‘Gene’ trees. Twelve 70% BS consensus trees summarised from parsimony and maximum likelihood phylogenetic analyses of individual non-recombinant regions (presented on two pages; A and B). Differences in topologies with respect to the positions of recombinant taxa are highlighted and the corresponding recombination events indicated. The trees are presented with the major groupings (PVY^C/NONPOT^, PVY^O^ and PVY^N-North America^, and PVY^N-Europe^), between which the recombinants switch, ordered consistently from top to bottom; these are also indicated by red, yellow, light blue and dark blue bars respectively. Where resolution of particular trees is too limited to retrieve these clades their membership – give or take recombinants – is assumed to be consistent with previous or subsequent trees.

**Figure 4:**
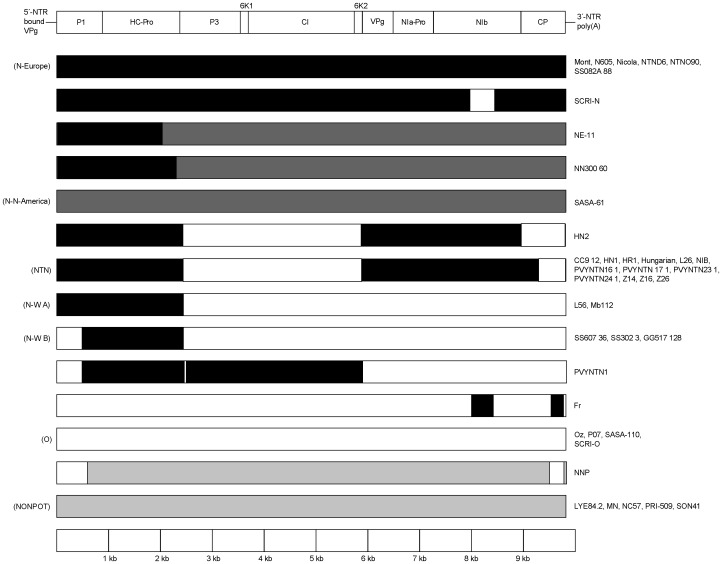
Recombination map of PVY genomes. Patterns of recombination between PVY strains PVY^O^ (white), PVY^N-North America^ (dark grey), PVY^N-Europe^ (black) and PVY^NONPOT^ (light grey) are illustrated.

A supermatrix was subsequently constructed in which recombinant sequences were split into multiple taxa using the ‘taxon duplication’ approach [31], [32]; equivalent to the ‘compatibility matrix’ output format implemented in RDP3 [48] (Dataset S2). Following this approach, taxa that exhibit conflicting phylogenetic positions according to different gene regions are duplicated in the matrix and the (different) conflicting gene regions re-coded as missing data for each duplicate. The resulting supermatrix can be analyzed using standard phylogenetic techniques to produce a single ‘multi-labelled’ tree in which conflicting taxa – e.g. putative hybrids or recombinants – are represented more than once. This approach has previously been applied to phylogenetic analyses of conflicting gene trees in various groups of flowering plants [57]–[59], including an extension in a coalescence framework ([60] under the assumption that reticulation could be discerned from coalescent stochasticity). To our knowledge, the approach has not previously been applied to multiple recombinants or whole genomes of viruses. In order to determine which non-contiguous genome regions should be combined as single taxa in the supermatrix we first identified homologous recombination patterns, on the basis of common breakpoints and/or (given the possibility for nested recombinants) monophyly in gene trees; and then identified shared phylogenetic signal across non-contiguous genome regions (i.e. those interrupted by recombinant regions) on the basis of gene tree topological congruence and the logical sequence of homologous recombination events (Fig. 2). Where the evidence for combining non-contiguous genome regions was equivocal, we excluded from the analyses the shorter regions from the taxa in question, recoding them as unknown in the matrix (Fig. 2).

### Phylogenetic Analyses

Phylogenetic analyses were performed under parsimony using PAUP* 4.0b10 [61] and under likelihood using RAxML [62]. Under parsimony, the following heuristic search options were employed: 500 random addition sequences (RAS) with tree bisection and reconnection (TBR) branch swapping saving a maximum of 25 trees of minimal length in each replicate. Clade support was estimated using 10,000 replicates of non-parametric bootstrapping each comprising a single RAS and TBR, saving a single tree in each replicate. RAxML analyses were performed using the CIPRES Science Gateway (http://www.phylo.org/portal2/) [63], [64], assuming a gamma model of rate heterogeneity. The supermatrix was analysed as above both with and without monophyly constraints on eight clades descendent from specific recombination events. This topological constraint represents the strong phylogenetic evidence of genome-scale processes that is further demonstrated by the monophyly of the clades in the separate analyses of non-recombinant gene regions. It may be important in preventing arbitrary groupings of closely related recombinant (duplicated) taxa that in the supermatrix do not share overlapping sequences. Under the tentative assumption of a reticulation scenario, phylogenetic networks were summarised using SplitsTree 4.12 [65] and Dendroscope 3 [66], 1) from the trees resulting from individual analyses of non-recombining regions and 2) from the single multi-labelled tree (in which recombinant taxa are represented more than once) resulting from analysis of the supermatrix. In both cases, nodes subject to <70% BS were first collapsed to form polytomies. Using Splitstree, consensus splits of trees were computed using the Consensus Network method [67] and splits were transformed into a reticulate network using the RECOMB2007 method [68]; cluster-based rooted networks were computed using Dendroscope.

**Figure 5:**
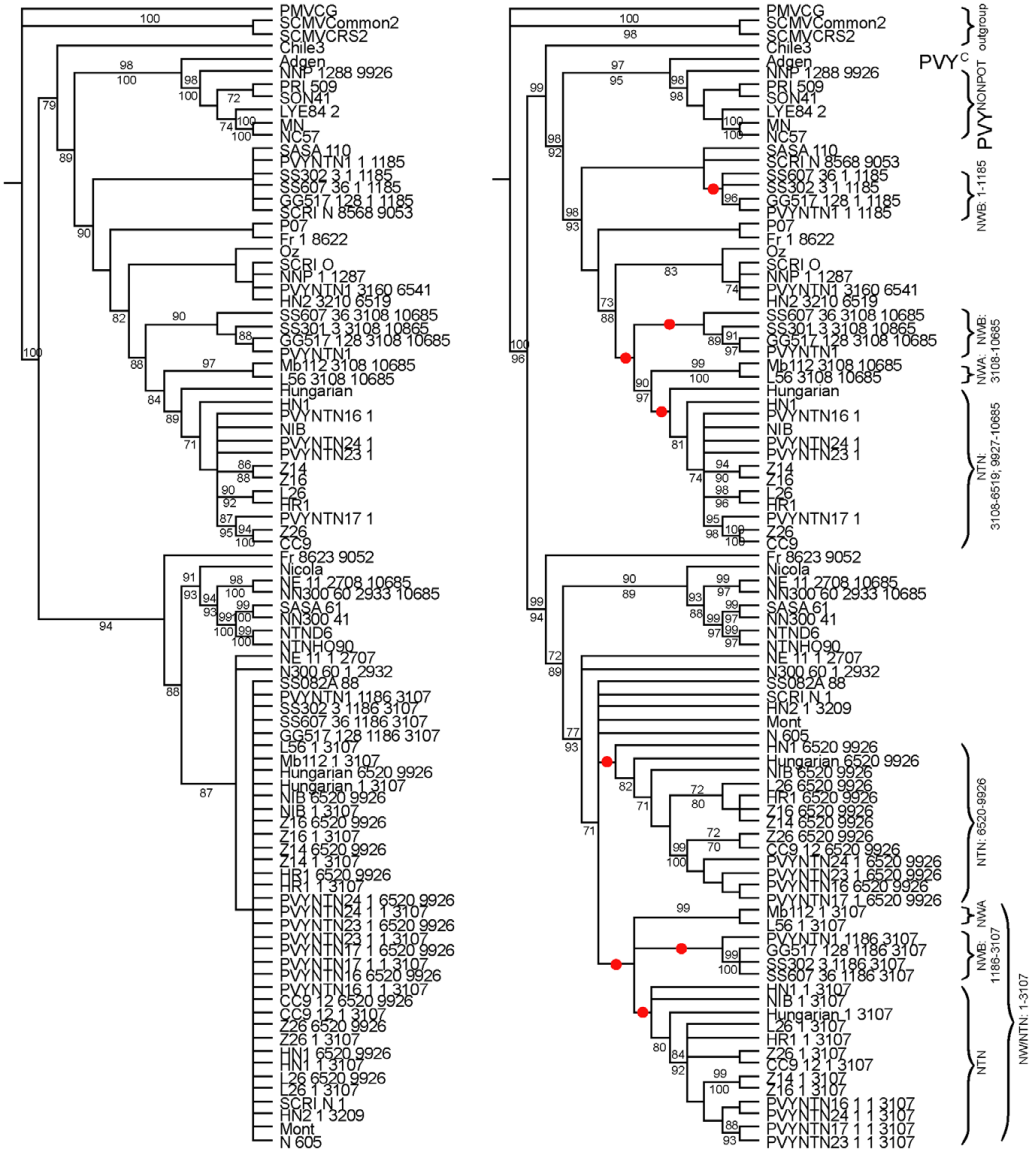
Supermatrix analysis. Parsimony strict consensus trees with bootstrap support above (parsimony) and below (ML) the branches are presented; a) without; and b) with the backbone monophyly constraint. Constrained nodes are indicated by red dots on the corresponding branches.

**Figure 6:**
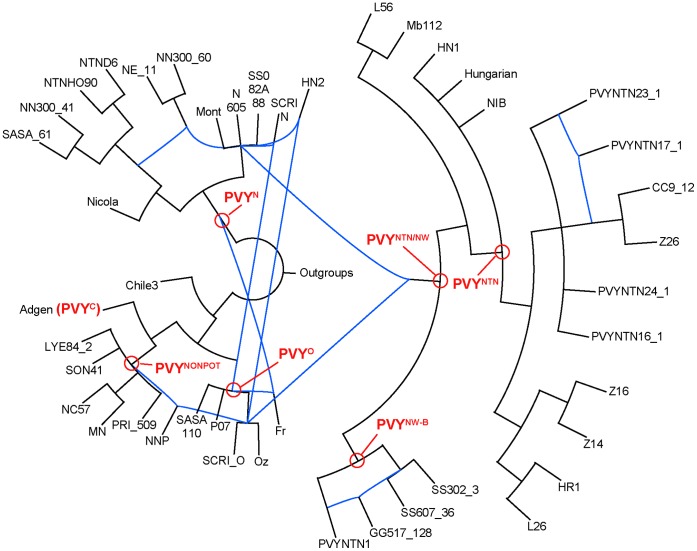
A reticulate phylogenetic hypothesis for PVY genomes. A rooted phylogenetic network, assuming that recombination (blue branches) represents reticulation events. The nodes representing the most recent common ancestors of PVY strains and recombinant clades are indicated with red circles and labels.

### Molecular Dating

Path-o-gen 1.3 [21] was used to investigate the ‘clocklikeness’ of the PVY phylogeny with an ML tree obtained using RAxML and asynchronous tip ages (as below). The supermatrix was analysed with the above topological constraint plus a monophyly constraint for the ingroup (to root the phylogeny) using BEAST 1.7.2 [21] on the CIPRES Science Gateway [64]. We applied fixed age constraints to tips to calibrate the rate of molecular evolution [69], [70]. For sequences not produced for this study this information was obtained from the authors of the original studies; where this was not possible the isolates were omitted from the analyses (Table S1). Age estimates are more precise when the range of tip age constraints spans a higher proportion of the total age of the group [70]. The total age range of the tips in these analyses spans the years 1982 to 2010 (i.e. 28 years), which is 35% of the 80 year putative timeframe for the observation of the disease in crops, but is likely to represent a much smaller proportion of the total age of PVY root node (which is unknown). Using the taxon duplication approach, the ages of recombinant isolates contribute to calibration in multiple branches of the tree (similar to the calibration of multiple homeologues in polyploids in [71]). We applied the general time reversible (GTR) substitution model with gamma distributed rates and a proportion of invariable sites. We applied strict clock (SC) and relaxed clock models, the latter assuming lognormal (LN) and exponential distributions (EX) of rates across the phylogeny, in order to assess the sensitivity of age estimates to assumptions regarding patterns of molecular rate variation, particularly given the potential (though, to our knowledge, as yet untested) impact of missing data. Under SC two MCMC runs of 10 million generations each were performed, each sampling trees every 1,000 generations. Under LN and EX three runs of 50 or 100 million generations each were performed, sampling trees every 10,000 generations. Shorter runs excluding the recombinant sequences were performed as a joint sensitivity test for the impact of calibrations and missing data. Removing taxa from the matrix might be expected to reduce the precision of age estimates due to the directly associated loss of information regarding rate calibration (i.e. both tip ages and molecular variation). However, should the wider confidence intervals of such age estimates not contain those inferred in the presence of significant proportions of missing data this might provide evidence for some form of bias. Likelihood and topological convergence and adequate sampling of the runs were confirmed using AWTY [72] and Tracer [73]. Randomisations of tip ages as a further test of the validity of rate estimates [74] were not feasible given the lengths of runs necessary to reach convergence. We note however that datasets shown to fail such tests are generally characterised by low levels of sequence variation and/or only produce precise age estimates when constrained by informative priors, e.g. on demographic parameters or on the age of the root [74]. Neither is the case here.

**Figure 7:**
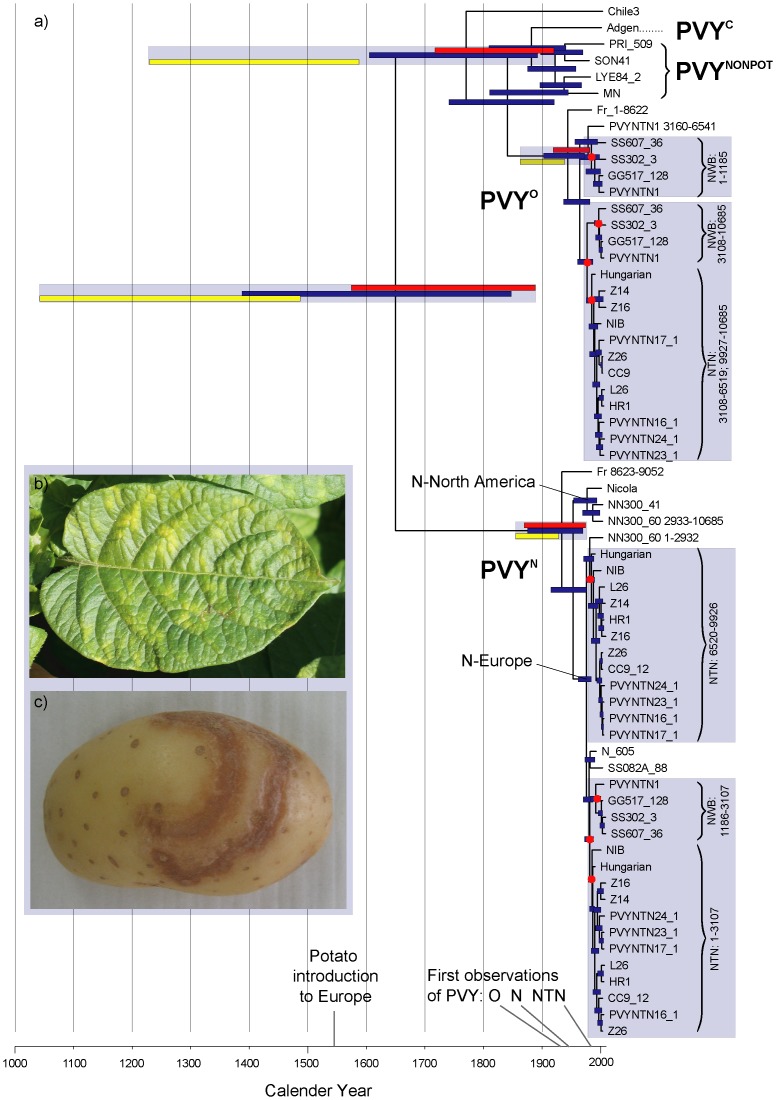
The recent, recombinant origins of Potato Virus Y genomes. The maximum clade credibility tree from the BEAST LN relaxed clock analysis is shown with error bars representing the 95% HPD of node ages according to LN (blue), EX (red) and SC (yellow) models. Recombinant strains such as pathogenic PVY^NTN^ and PVY^NW-B^ are represented as multiple taxa, each representing a subset of the alignment (as indicated) with distinct phylogenetic signal. Topology constrained nodes are indicated with red dots. Inset: a potato leaf showing mosaic patterns and tuber with potato tuber necrotic ring disease resulting from PVY infection.

**Table 1:** The ages of major strains of PVY.

Clade	LN	EX	SC
**PVY**	619-161	436-123	970-525
**N**	133-39	137-33	152-79
**N-Europe**	47-24	48-23	98-51
**N-N America**	55-15	47-12	55-31
**(Chile3, O, C, NONPOT)**	403-116	294-91	778-419
**(O, C, NONPOT)**	267-88	205-71	525-287
**(C, NONPOT)**	199-67	153-57	445-244
**O**	106-36	93-30	144-69
**NONPOT**	133-51	107-45	365-201

Age estimates (95% HPD; years before present) under relaxed clock lognormal (LN) and exponential (EX), and strict clock (SC), models using the taxon duplication supermatrix approach.

**Table 2:** The ages of recombination events in PVY.

Clade/recombination	Crown age (years; LN/EX/SC)	Stem age (years; LN/EX/SC)
NW/NTN 1-3107	36-**21**/34-**20**/47-**27**	**38**-22/**37**-21/**48**-29
NW/NTN 3108-10654	48-21/42-20/64-35	72-27/62-23/79-42
NTN 1-3107	30-**19**/29-**19**/41-**25**	**36**-21/**34**-20/**47**-27
NTN 3108-6519, 9927-10654	33-19/31-18/51-28	48-21/42-20/64-35
NTN 6520-9926	33-19/32-19/44-25	37-20/37-19/48-27
NWB 1-1185	44-10/35-8/73-24	52-14/43-10/145-69
NWB 1186-3107	26-9/25-7/35-17	**36**-21/**34**-20/**47**-27
NWB 3108-10654	19-**7**/23-**6**/19-**10**	48-21/42-20/64-35
PVYNTN1 3160-6541		52-14; 26-8; **21**-6/43-10; **17**-5; 25-7/38-10; 79-42; **35**-17
NN300-60		39-10; **37**-20/**32**-9; 37-19/98-51; **44**-25
FR		**106**-36; 133-39/**93**-30, 137-33/114-64; **112**-56

Minimum and maximum age estimates for the multiple recombination events leading to the origins of of PVY^NW^/^NTN^ and maximum age estimates for other recombination events. The most recent bounds of the crown node age of a recombinant clade (i.e. the Most Recent Common Ancestor [mrca] of the recombinants analysed) represents the most recent possible age for that recombination event. The oldest bounds of the stem node age (i.e. that of the node subtending the crown node, representing the mrca of the clade and its sister group) correspondingly represents the oldest possible age for the recombination event. ‘Crown ages’ for recombinant clades represented multiple times in the multi-labelled tree are directly comparable, and should be expected to be the same, give or take margins of error. ‘Stem ages’ by contrast are dependent on taxon sampling outside the crown group and thus should be expected to vary, with some stem node ages representing a greater overestimation of the age of the recombination event than others. Therefore the oldest possible age for the recombination event can be interpreted from the older bounds of the *most recent* of the stem node age estimates. The minimum and maximum ages are represented in bold type.

## Results

### Recombination Breakpoints and Homologous Recombination Patterns

We analysed 44 PVY genomes with recombination detection software in order to identify recombinant isolates and locate the recombinant regions of their genomes. Just 19 of these isolates can be regarded as non-recombinant. Five are single recombinants and 20 show two or more recombination events. We located 17 breakpoints and inferred phylogenetic trees for the corresponding sequence of non-recombining regions under parsimony and Maximum Likelihood (ML) to confirm topological differences between trees (Fig. 3). We then identified homologous recombination patterns on the basis of common breakpoints and/or (given the possibility for nested recombinants) monophyly in gene trees. Both were evident for PVY^NTN^ (18 isolates) and PVY^N-W^ B-type (four isolates) separately and for PVY^N-W^ A- and B-types plus PVY^NTN^ together (Figs. 2 and 3). A further 10 recombination events (of 13 in total) were represented by single isolates (Table S2). Recombinant regions, the strains/isolates that exhibit them and the genome type (PVY^NONPOT^: a strain found in various plants other than potatoes; PVY^O^ or PVY^N^) involved are illustrated in Fig. 4.

### Supermatrix Construction

In order to simultaneously infer the phylogenetic relationships of both non-recombinant and recombinant whole genomes we identified shared phylogenetic signal across non-contiguous genome regions (i.e. those interrupted by recombinant regions) on the basis of gene tree topological congruence and the logical sequence of homologous recombination events (Figs. 2 and 3). On this basis, the whole genome sequences of single recombinants were subdivided between two taxa each in the matrix, double recombinants PVY^N-W^ B-type and PVY^NTN^ between three, and triple recombinant PVYNTN1 between four; each taxon representing a subset of the alignment with distinct phylogenetic signal with the rest of the alignment coded as missing data (Fig. 2). In the case of double recombinants Fr and NNP, the phylogenetic signals were not sufficiently strong to discern congruence and conflict, and ancestral-type single recombinants are unknown. Recombinant regions 9052–10654 and 10172–10654 of Fr and NNP respectively were therefore excluded from further analyses by means of recoding as missing data (Fig. 2).

### Phylogenetic Inference

Phylogenetic analyses were performed on the resulting supermatrix under parsimony and ML. Of 9,723 characters included in the analyses (reduced from an alignment of 10,685 with outgroups), 5,683 were variable and 4,267 parsimony informative. In order to avoid potential loss of phylogenetic resolution between taxa with entirely non-overlapping sequences a topological constraint was designed to enforce the monophyly of each clade of homologous recombinants. This corresponded to eight nodes of a total of 89 given a 90 taxa bifurcating tree (Fig. 5). The results were entirely congruent irrespective of whether this topological constraint was applied or not, but the constrained analysis resulted in considerably higher resolution both within and between constrained clades without leading to an increase in shortest tree length (Fig. 5). Networks summarised from the individual gene trees (non-recombinant regions separately; Fig. S1 A) and the multi-labelled tree (resulting from analysis of the supermatrix; Fig. 6, Fig. S1 B) were broadly comparable, but the former considerably more complex. In both cases network structure was revealed within monophyletic PVY^NTN^ and PVY^N-W^ B clades.

### Molecular Dating

Path-o-gen was used to calculate a regression of root-to-tip distances against dates of sampling. The slope of the regression (representing the rate) was 0.00237; with correlation coefficient (variation in rate) 0.1885; R squared 0.0355; and residual mean squared 0.0073; indicating deviation from a strict molecular clock. In order to infer simultaneously phylogenetic relationships and the ages of clades and recombination events we analysed the supermatrix under Bayesian inference with both strict and relaxed clock models, including only the 28 (of 44) PVY isolates for which we could obtain accurate asynchronous sampling dates. Tree samples from independent Bayesian runs of the supermatrix under each model showed consistent and stable posterior probability (PP) clade support and effective sampling sizes for model parameters >200. The maximum clade credibility tree of the LN analysis is illustrated in Fig. 7; nodes subject to ≥0.95 PP were consistent with those ≥70% BS inferred under parsimony and ML (data not shown). In general, strict clock (SC) based estimates for deeper nodes are older than those based on either lognormal (LN) or exponential (EX) models, but the discrepancy is smaller for more recent nodes. The 95% highest posterior density interval (95% HPD) for the clock/mean rate were 0.0001–0.0003 (SC); 0.0003–0.0012 (LN); and 0.0005–0.0015 (EX). The 95% HPD for the standard deviation of the LN relaxed clock was 0.6128–1.0086, which as it does not include zero further indicates the rejection of the strict molecular clock. The 95% HPD for the crown node of PVY dates to 619-161 (LN)/436-123 (EX)/970-525 (SC) years ago. Crown nodes ages of PVY^O^, PVY^N^ and PVY^NONPOT^ are similar according to the different methods, falling between around 150 and 30 years (although PVY^NONPOT^ is older under the SC model: 365-201 years; Table 1). Excluding the recombinant isolates (leaving just 13 of 66 taxa in the supermatrix) resulted in much broader but overlapping age ranges, e.g. for the PVY crown node: 4,738-84 (LN); 3,498-58 (EX); and 133,080-801 (SC). Hence the confidence intervals extended considerably further back in time (there was no prior constraint for the age of the root node in any of the analyses), but with the exception of the SC model also increased towards the present (which is by definition constrained by the ages assigned to the tips). In the relaxed clock results based on full taxon sampling with missing data there was thus no obvious bias towards either older or more recent age estimates. The range of crown and stem node age estimates for recombinant clades (i.e. PVY^N-W^/PVY^NTN^, PVY^NTN^ and PVY^N-W^ B) place the corresponding recombination events between 48 and 20; 47 and 19; and 47 and 6 years ago, respectively (Table 2). Recombination events represented by single isolates have stem ages in years as follows: PVYNTN1: 35-17; NN300_60: 44-32; and Fr: 112-93.

## Discussion

The most serious consequences of PVY infection are yield reduction and PTNRD. Specific genes are currently under investigation for their potential to cause pathologies [1], [5], [6], [16] but the symptoms are generally worse in the recombinant strains PVY^NTN^ and PVY^NW^ and recombination has also been directly implicated as a cause of pathogenicity [36]. Our results show that recombination is widespread amongst both highly pathogenic and less pathogenic PVY strains. We recovered essentially the same breakpoints identified for individual isolates in previous work (suggesting that our recombination detection approach was not overly conservative), as well as identifying a novel recombination pattern in NN300_60, a South African isolate similar to the previously described NE-11 [28]. The resulting phylogeny shows the major groupings of PVY strains [24], [26]–[29] and qualifies the phylogenetic affinities of the isolate Chile3 (apparently not the sister group to PVY, contra [24]), whilst simultaneously identifying the multiple phylogenetic relationships of the recombinants.

Our results confirm the single origins of recombinant PVY^NTN^ and PVY^NW^ strains, whilst at the same time providing evidence for recombination events within those strains. Both the original recombination events and some of those occurring subsequently must be associated with more or less identical breakpoints. This phenomenon has been reported for other viruses, such as begomoviruses, which have been shown to recombine at non-random breakpoints [13], due at least in part to selection [75]. The extreme consequence of such a process would be where structural genes in viruses represent ‘functionally interchangeable modules with effectively independent evolution’ [14]. Our results nevertheless imply tractable sequences of recombination events within an otherwise effectively tree-like underlying phylogeny. With the supermatrix approach, the phylogenetic affinities of even relatively short (non-recombinant) genome regions can be assessed within the strong phylogenetic ‘scaffold’ provided by the other data [31], [76]. The improved overall resolution that we obtained for the PVY phylogeny using the taxon duplication-based supermatrix compared to the separate analyses of non-recombinant genome regions is reflected in a simpler network with far fewer alternative connections between recombinants and non-recombinants (Fig. S1).

Further investigation of our approach (and of supermatrix analyses in general) should address the potential impact of missing data on age estimation. Although no obvious bias was apparent here (with the exception of the arguably inappropriate SC model), the power of the assessment was limited by the small number of non-recombinant genomes analysed. Assuming that the models implemented in BEAST do adequately reflect the uncertainty involved, the advantages of our approach are clear: by including both non-recombinant and recombinant isolates in single molecular dating analyses in this manner we were able to estimate the ages of both clades and recombination events between them, with improved precision relative to analysis of non-recombinants alone.

The potato, *Solanum tuberosum* L., originated in the New World and its centre of diversity is in the Andes. All cultivated varieties descend from a single domestication [77], with the first archaeological evidence of potato use dating to ca. 750 BC in Peru and the first likely cultivation of potatoes dating to 400 AD [78]. However, our age estimates for the most recent common ancestor (mrca) of PVY correspond more closely to the timing of the first introductions of potatoes to Europe (between 1540 and 1565 to Spain and in 1565 to Britain [79]), consistent with the recent origins inferred for various plant viruses [80]. The age estimates could indicate origin either in the New World before the European introduction, or thereafter, but given that our oldest age estimates were produced under the SC model, which appears less appropriate for this data, the latter seems more plausible. Age estimates for deeper nodes in the phylogeny are imprecise, which is inevitable given the relatively recent asynchronous tip ages used for the molecular clock calibration. However, our calibration is independent of any assumptions regarding correlations of particular phylogenetic relationships with historical isolation and outbreak events (as used by [30] in their study of potyviruses). This logical independence is crucial given our aim to test exactly these kinds of hypotheses. Despite the wide confidence intervals, even the oldest estimates for the ages of the crown nodes of PVY^O^ and PVY^N^ fall within the last c. 150 years. This places the timeframe of the radiation of PVY clades known from potato crops, as well as all known recombination events between those clades, well within that of modern potato cultivation.

In fact, most recombination events inferred here are conspicuously recent. PVY^N-W^ A-type descends from a common recombination between PVY^O^ and PVY^N^ which dates to 48-20 years ago, and PVY^NTN^ and PVY^N-W^ B-type are the results of two subsequent recombination events that we date to between 47 and 19 years ago and 47 and 6 years ago, respectively. These ages are consistent with the first observations of symptoms associated with specific strains in potato crops. PVY strains isolated from non-potato hosts are restricted to the PVY^NON-POT^ clade and isolate Chile3 [24] and gene flow (in the form of recombination) between these and other PVY clades appears to be rare compared to that between and within PVY^O^ and PVY^N^. Overall, these suggest that the strains of PVY currently infecting crops have evolved as specialists of potato cultivars and not, as might have been the case, by lateral transfer from other hosts. They are consistent with recent (recombinant) origins of pathogenic strains of PVY within modern potato crops. Given the age estimates, these may have been particularly associated with increased 20^th^ Century international trade; there is no evidence for earlier recombination between the major PVY strains.

Advances in transport have inevitably led to the increasingly rapid distribution of material infected with different strains of PVY^O^ and PVY^N^. It is clear from the age estimates presented here, as well as the high genetic diversity of PVY strains found in individual countries such as South Africa [33], that measures to control such movement were implemented subsequent to the origin and spread of the most damaging PVY recombinant strains. Our results illustrate how recombination between both distantly and closely related strains of PVY has contributed to the origin of pathogenic strains such as PVY^NTN^. They also provide evidence for ongoing recombination within PVY^NTN^, as might be expected from patterns observed in other viruses [11]–[14]. This process is a cause for concern in the context of disease prevention, as it could facilitate combinations of sequence variants that increase virus fitness, for example by improving transmission. For crop plants, it is possible at least in principle to reduce the spread of diseased material by stringent testing as part of national certification schemes and monitoring of imports. Our results serve to further highlight the importance of such efforts.

## Supporting Information

Figure S1 **Phylogenetic networks summarised using SplitsTree a) from the 12 70% BS consensus trees in** **Fig. 3** **; b) from a single 70% BS consensus of the multi-labelled tree in** **Fig. 5** **b.** Nodes recovered in one network but contradicted in the other are indicated with red dots on the corresponding branches. Major PVY strains and recombinant clades are indicated.
(TIF)

Table S1 **Accessions details, including details regarding tip dates used in BEAST analyses and from who the information was obtained.**
(DOC)

Table S2 **Summary of break points identified in the genomes of the isolates analysed in this study.**
(DOC)

Dataset S1 **Standard alignment of sequences used in this study.**
(NEX)

Dataset S2 **Supermatrix used in this study.**
(NEX)

## References

[1] McDonald JD, Singh RP (1996) Host range, symptomology, and serology of isolates of potato virus Y (PVY) that share properties with both the PVY^N^ and PVY^O^ strain groups. The American Potato Journal 73: 309–313.

[2] Ward CW, Shukla DD (1991) Taxonomy of potyviruses: current problems and some solutions. Intervirology 32: 269–296. 1657820 10.1159/000150211

[3] Valkonen JP (2007) Viruses: economical losses and biotechnological potential. In: Vreugdenhil D, Bradshaw J, Gebhardt C, Govers F, Taylor M et al.., editors. Potato biology and biotechnology: Advances and perspectives. Amsterdam: Elsevier. 619–641.

[4] Warren M, Krüger K, Schoeman AS (2005) Potato virus Y (PVY) and potato leaf roll virus (PLRV): Literature review for potatoes South Africa. Department of Zoology and Entomology, Faculty of Natural and Agricultural Sciences, University of Pretoria.

[5] Le Romancer M, Kerlan C, Nedellec M (1994) Biological characterisation of various geographical isolates of potato virus Y inducing superficial necrosis on potato tubers. Plant Pathology 43: 138–144.

[6] Boonham N, Walsh K, Hims M, Preston S, North J, et al (2002) Biological and sequence comparisons of Potato virus Y isolates associated with potato tuber necrotic ringspot disease. Plant Pathology 51: 117–126.

[7] Salaman RN (1930) Virus diseases of potato: Streak. Nature 126: 241.

[8] Smith KM (1931) Composite nature of certain potato viruses of the mosaic group. Nature 127: 702.

[9] Orlando A, Silberschmidt K (1945) Estudos sôbre a transmissao da doenca de Solanaceas “Necroses das Nervuras”. por afïdios, e algumas relacoes entre esse virus e o seu principle inseto vector. Arquivos do Instituto Biológico 6: 133–152.

[10] Crosslin JM, Hamm PB, Shiel PJ, Hane DC, Brown CR, et al (2005) Serological and molecular detection of tobacco veinal necrosis isolates of Potato virus Y (PVY^N^) from potatoes grown in the western United States. American Journal of Potato Research 82: 263–269.

[11] Castro-Nallar E, Pérez-Losada M, Burton GF, Crandall KA (2012) The evolution of HIV: Inferences using phylogenetics. Molecular Phylogenetics and Evolution 62: 777–792. 22138161 10.1016/j.ympev.2011.11.019 PMC3258026

[12] Lole KS, Bollinger RC, Paranjape RS, Gadkari D, Kulkarni SS, et al (1999) Full-Length Human Immunodeficiency Virus Type 1 Genomes from Subtype C-Infected Seroconverters in India, with Evidence of Intersubtype Recombination. J Virol 73: 152–160. 9847317 10.1128/jvi.73.1.152-160.1999 PMC103818

[13] Lefeuvre P, Martin DP, Hoareau M, Naze F, Delatte H, et al (2007) Begomovirus ‘melting pot’ in the south-west Indian Ocean islands: molecular diversity and evolution through recombination. J Gen Virol 88: 3458–3468. 18024917 10.1099/vir.0.83252-0

[14] Heath L, van der Walt E, Varsani A, Martin DP (2006) Recombination Patterns in Aphthoviruses Mirror Those Found in Other Picornaviruses. J Virol 80: 11827–11832. 16971423 10.1128/JVI.01100-06 PMC1642601

[15] Tan Z, Wada Y, Chen J, Ohshima K (2004) Inter- and intralineage recombinants are common in natural populations of Turnip mosaic virus. Journal of General Virology 85: 2683–2696. 15302962 10.1099/vir.0.80124-0

[16] Beczner L, Horvath J, Romhanyi I, Forster H (1984) Studies on the etiology of tuber necrotic ringspot disease in potato. Potato Research 27: 339–352.

[17] Chrzanowska M (1991) New isolates of the necrotic strain of potato virus Y (PVY^N^) found recently in Poland. Potato Research 34: 179–182.

[18] Singh RP, Valkonen JPT, Gray SM, Boonham N, Jones RAC, et al (2008) Discussion paper: The naming of *Potato virus Y* strains infecting potato. Archives of Virology 153: 1–13. 17943395 10.1007/s00705-007-1059-1

[19] Rahman MS, Akanda AM (2009) Performance of seed potato produced from sprout cutting, stem cutting and conventional tuber against PVY and PLRV. Bangladesh Journal of Agricultural Research 34: 609–622.

[20] Blanchard A, Rolland M, Delaunay A, Jacqout E (2008) An international organization to improve knowledge on Potato virus Y. In: Tennant P, Benkeblia N, Blanchard A, Rolland M, Delaunay A et al., editors. Potato II Fruit, Vegetable and Cereal Science and Biotechnology 3: 6–9.

[21] Drummond AJ, Rambaut A (2007) BEAST: Bayesian evolutionary analysis by sampling trees. BMC Evolutionary Biology 7: 214. 17996036 10.1186/1471-2148-7-214 PMC2247476

[22] Drake JW (1993) Rates of spontaneous mutation among RNA viruses. Proceedings of the National Academy of Sciences 90: 4171–4175. 10.1073/pnas.90.9.4171 PMC46468 8387212

[23] Cuevas JM, Delaunay A, Visser JC, Bellstedt DU, Jacquot E, et al.. (2012) Phylogeography and molecular evolution of Potato virus Y. PLoS ONE. 10.1371/journal.pone.0037853 PMC3360008 22655074

[24] Moury B (2010) A new lineage sheds light on the evolutionary history of *Potato virus Y*. Molecular Plant Pathology. 11: 161–168. 10.1111/j.1364-3703.2009.00573.x PMC6640215 20078785

[25] Galvino-Costa SBF, Dos Reis Figueira A, Camargos VV, Geraldino PS, Hu XJ, et al.. (2011) A novel type of Potato virus Y recombinant genome, determined for the genetic strain PVYE. Plant Pathology: 1–11.

[26] Ogawa T, Tomitaka Y, Nakagawa A, Ohshima K (2007) Genetic structure of a population of Potato virus Y inducing potato tuber necrotic ringspot disease in Japan; comparison with North American and European populations. Virus Research 131: 199–212. 18029044 10.1016/j.virusres.2007.09.010

[27] Hu X, Meacham T, Ewing L, Gray SM, Karasev AV (2009) A novel recombinant strain of Potato virus Y suggests a new viral genetic determinant of vein necrosis in tobacco. Virus Research 143: 68–76. 19463723 10.1016/j.virusres.2009.03.008

[28] Lorenzen JH, Nolte P, Martin D, Pasche JS, Gudmestad NC (2008) NE-11 represents a new strain variant class of Potato virus Y. Archives of Virology. 153: 517–525. 10.1007/s00705-007-0030-5 18193154

[29] Karasev AV, Hu X, Brown CJ, Kerlan C, Nikolaeva OV, et al (2011) Genetic diversity of the Ordinary Strain of Potato virus Y (PVY) and origin of recombinant PVY strains. Phytopathology 101: 778–785. 21675922 10.1094/PHYTO-10-10-0284 PMC3251920

[30] Gibbs AJ, Ohshima K, Phillips MJ, Gibbs MJ (2008) The Prehistory of Potyviruses: Their Initial Radiation Was during the Dawn of Agriculture. PLoS ONE 3: e2523. 18575612 10.1371/journal.pone.0002523 PMC2429970

[31] Pirie MD, Humphreys AM, Galley C, Barker NP, Verboom GA, et al (2008) A novel supermatrix approach improves resolution of phylogenetic relationships in a comprehensive sample of danthonioid grasses. Molecular Phylogenetics and Evolution 48: 1106–1119. 18599319 10.1016/j.ympev.2008.05.030

[32] Pirie MD, Humphreys AM, Barker NP, Linder HP (2009) Reticulation, data combination, and inferring evolutionary history: an example from Danthonioideae (Poaceae). Systematic Biology 58: 612–628. 20525613 10.1093/sysbio/syp068

[33] Visser JC, Bellstedt DU (2009) An assessment of molecular variability and recombination patterns in South African isolates of Potato virus Y. Archives of Virology. 154: 1891–1900. 10.1007/s00705-009-0525-3 19862472

[34] Lorenzen JH, Piche LM, Gudmestad NC, Meacham T, Shiel P (2006) A Multiplex PCR assay to characterize Potato virus Y Isolates and identify strain mixtures. Plant Disease 90: 935–940. 10.1094/PD-90-0935 30781033

[35] Bellstedt DU, Pirie MD, Visser JC, de Villiers MJ, Gehrke B (2010) A rapid and inexpensive method for the direct PCR amplification of DNA from plants. Am J Bot 97: e65–68. 21616856 10.3732/ajb.1000181

[36] Barker H, McGeachy KD, Toplak N, Gruden K, Žel J, et al (2009) Comparison of genome sequence of PVY isolates with biological properties. American Journal of Potato Research 86: 227–238.

[37] Fanigliulo A, Comes S, Pacella R, Harrach B, Martin DP, et al (2005) Characterisation of Potato virus Y nnp strain inducing veinal necrosis in pepper: a naturally occurring recombinant strain of PVY. Archives of Virology 150: 709–720. 15592887 10.1007/s00705-004-0449-x

[38] Fellers JP, Tremblay D, Handest MF, Lommel SA (2002) The Potato virus Y M(S)N(R) NIb-replicase is the elicitor of a veinal necrosis-hypersensitive response in root knot nematode resistant tobacco. Molecular Plant Pathology 3: 145–152. 20569320 10.1046/j.1364-3703.2002.00106.x

[39] Hu X, He C, Xiao Y, Xiong X, Nie X (2009) Molecular characterization and detection of recombinant isolates of potato virus Y from China. Archives of Virology 154: 1303–1312. 19597695 10.1007/s00705-009-0448-z

[40] Jakab G, Droz EBG, Baulcombe D, Malnoe P (1997) Infectious in vivo and in vitro transcripts from a full-length cDNA clone of PVY-N605, a Swiss necrotic isolate of potato virus Y. Journal of General Virology. 78: 3141–3145. 10.1099/0022-1317-78-12-3141 9400962

[41] Moury B, Morel C, Johansen E, Jacquemond M (2002) Evidence for diversifying selection in Potato virus Y and in the coat protein of other potyviruses. Journal of General Virology 83: 2563–2573. 12237440 10.1099/0022-1317-83-10-2563

[42] Nie X, Singh RP, Singh M (2004) Molecular and pathological characterization of N:O isolates of the Potato virus Y from Manitoba, Canada. Canadian Journal of Plant Pathology 26: 573–583.

[43] Robaglia C, Durand-Tardif M, Tronchet M, Boudazin G, Astier-Manifacier S, et al (1989) Nucleotide sequence of Potato virus Y (Strain N) genomic RNA. Journal of General Virology 70: 935–947. 2732709 10.1099/0022-1317-70-4-935

[44] Schubert J, Fomitcheva V, Sztangret-Wisniewska J (2007) Differentiation of Potato virus Y strains using improved sets of diagnostic PCR-primers. Journal of Virological Methods 140. 10.1016/j.jviromet.2006.10.017 17182113

[45] Singh M, Singh RP (1996) Nucleotide sequence and genome organization of a Canadian isolate of the common strain of potato virus Y (PVYO). Canadian Journal of Plant Pathology 18: 209–214.

[46] Thole V, Dalmay T, Burgyan J, Balazs E (1993) Cloning and sequencing of potato virus Y (Hungarian isolate) genomic RNA. Gene 123: 149–156. 8428653 10.1016/0378-1119(93)90118-m

[47] Hall TA (1999) BioEdit: a user-friendly biological sequence alignment editor and analysis program for Windows 95/98/NT; 95–98.

[48] Martin DP, Lemey P, Lott M, Moulton V, Posada D, et al (2010) RDP3: a flexible and fast computer program for analyzing recombination. Bioinformatics 26: 2462–2463. 20798170 10.1093/bioinformatics/btq467 PMC2944210

[49] Martin D, Rybicki E (2000) RDP: detection of recombination amongst aligned sequences. Bioinformatics 16: 562–563. 10980155 10.1093/bioinformatics/16.6.562

[50] Padidam M, Sawyer S, Fauquet CM (1999) Possible emergence of new geminiviruses by frequent recombination. Virology 265: 218–225. 10600594 10.1006/viro.1999.0056

[51] Sawyer S (1989) Statistical tests for detecting gene conversion. Molecular Biology and Evolution 6: 526–538. 2677599 10.1093/oxfordjournals.molbev.a040567

[52] Maynard Smith J (1992) Analyzing the mosaic structure of genes. Journal of Molecular Evolution 34: 126–129. 1556748 10.1007/BF00182389

[53] Posada D (2001) Unveiling the molecular clock in the presence of recombination. Molecular Biology and Evolution 18: 1976–1978. 11557803 10.1093/oxfordjournals.molbev.a003738

[54] Martin DP, Posada D, Crandall KA, Williamson C (2005) A modified bootscan algorithm for automated identification of recombinant sequences and recombination breakpoints. AIDS Research and Human Retroviruses 21: 98–102. 15665649 10.1089/aid.2005.21.98

[55] Salminen MO, Carr JK, Burke DS, McCutchan FE (1995) Identification of breakpoints in intergenotypic recombinants of HIV type 1 by BOOTSCANning. AIDS Research and Human Retroviruses 11: 1423–1425. 8573403 10.1089/aid.1995.11.1423

[56] Gibbs MJ, Armstrong JS, Gibbs AJ (2000) Sister-Scanning: a Monte Carlo procedure for assessing signals in recombinant sequences. Bioinformatics 16: 573–582. 11038328 10.1093/bioinformatics/16.7.573

[57] Pelser PB, Kennedy AH, Tepe EJ, Shidler JB, Nordenstam B, et al (2010) Patterns and causes of incongruence between plastid and nuclear Senecioneae (Asteraceae) phylogenies. American Journal of Botany 97: 856–873. 21622451 10.3732/ajb.0900287

[58] Antonelli A, Humphreys AM, Lee WG, Linder HP (2010) Absence of mammals and the evolution of New Zealand grasses. Proceedings of the Royal Society B: Biological Sciences 278: 675–701. 20826486 10.1098/rspb.2010.1145 PMC3030840

[59] Humphreys AM, Antonelli A, Pirie MD, Linder HP (2011) Ecology and evolution of the diaspore “burial syndrome”. Evolution 65: 1163–1180. 21062276 10.1111/j.1558-5646.2010.01184.x

[60] Blanco-Pastor JL, Vargas P, Pfeil BE (2012) Coalescent Simulations Reveal Hybridization and Incomplete Lineage Sorting in Mediterranean *Linaria* . PLoS ONE 7: e39089. 22768061 10.1371/journal.pone.0039089 PMC3387178

[61] Swofford DL (2003) PAUP*: Phylogenetic Analysis Using Parsimony (*and Other Methods), version 4. Sunderland, Mass.: Sinauer Associates.

[62] Stamatakis A (2006) RAxML-VI-HPC: maximum likelihood-based phylogenetic analyses with thousands of taxa and mixed models. Bioinformatics 22: 2688–2690. 16928733 10.1093/bioinformatics/btl446

[63] Stamatakis A, Hoover P, Rougemont J (2008) A Rapid Bootstrap Algorithm for the RAxML Web Servers. Systematic Biology 57: 758–771. 18853362 10.1080/10635150802429642

[64] Miller MA, Pfeiffer W, Schwartz T (2010) Creating the CIPRES Science Gateway for inference of large phylogenetic trees; New Orleans, LA. 1–8.

[65] Huson DH, Bryant D (2006) Application of phylogenetic networks in evolutionary studies Molecular Biology and Evolution. 23: 254–267. 10.1093/molbev/msj030 16221896

[66] Huson D, Richter D, Rausch C, Dezulian T, Franz M, et al (2007) Dendroscope: An interactive viewer for large phylogenetic trees. BMC Bioinformatics 8: 460–460. 18034891 10.1186/1471-2105-8-460 PMC2216043

[67] Holland BR, Benthin S, Lockhart PJ, Moulton V, Huber KT (2008) Using supernetworks to distinguish hybridization from lineage-sorting. BMC Evolutionary Biology 8: 202–202. 18625077 10.1186/1471-2148-8-202 PMC2500029

[68] Huson DH, Klöpper TH (2007) Beyond Galled Trees - Decomposition and Computation of Galled Networks. Research in Computational Molecular Biology. In: Speed T, Huang H, Huson DH, Klöpper TH, editors. Lecture Notes in Computer Science. Berlin, Heidelberg: Springer. 211–225.

[69] Drummond AJ, Ho SYW, Phillips MJ, Rambaut A (2006) Relaxed Phylogenetics and Dating with Confidence. PLoS Biology 4: e88. 16683862 10.1371/journal.pbio.0040088 PMC1395354

[70] Lemey P, Rambaut A, Drummond AJ, Suchard MA (2009) Bayesian Phylogeography Finds Its Roots. PLoS Computational Biology 5: e1000520. 19779555 10.1371/journal.pcbi.1000520 PMC2740835

[71] Marcussen T, Jakobsen KS, Danihelka J, Ballard HE, Blaxland K, et al (2012) Inferring species networks from gene trees in high-polyploid North American and Hawaiian violets (Viola, Violaceae). Systematic Biology 61: 107–126. 21918178 10.1093/sysbio/syr096 PMC3243738

[72] Wilgenbusch JC, Warren DL, Swofford DL (2004) AWTY: a system for graphical exploration of MCMC convergence in Bayesian phylogenetic inference. Available: http://king2.scs.fsu.edu/CEBProjects/awty/awty_start.php. Accessed 2012 Oct 29. 10.1093/bioinformatics/btm388 17766271

[73] Rambaut A, Drummond AJ (2003) Tracer v. 1.5. Available: http://tree.bio.ed.ac.uk/software/tracer/. Accessed 2012 Oct 29.

[74] Ho SYW, Lanfear R, Phillips MJ, Barnes I, Thomas JA, et al (2011) Bayesian Estimation of Substitution Rates from Ancient DNA Sequences with Low Information Content. Systematic Biology 60: 366–375. 21296909 10.1093/sysbio/syq099

[75] Lefeuvre P, Lett JM, Varsani A, Martin DP (2009) Widely Conserved Recombination Patterns among Single-Stranded DNA Viruses. The Journal of Virology 83: 2697–2707. 19116260 10.1128/JVI.02152-08 PMC2648288

[76] Wiens JJ (2006) Missing data and the design of phylogenetic analysis. Journal of Biomedical Informatics 39: 34–42. 15922672 10.1016/j.jbi.2005.04.001

[77] Spooner DM, McLean K, Ramsay G, Waugh R, Bryan GJ (2005) A single domestication for potato based on multilocus amplified fragment length polymorphism genotyping. Proceedings of the National Academy of Sciences of the United States of America 102: 14694–14699. 16203994 10.1073/pnas.0507400102 PMC1253605

[78] Towle MA (1961) The Ethnobotany of Pre-Columbian Peru. Chicago: Aldine.

[79] Brücher H (1975) Domestikation und Migration von *Solanum tuberosum* L. Genetic Resources and Crop Evolution. 23: 11–74.

[80] Gibbs AJ, Fargette D, García-Arenal F, Gibbs MJ (2010) Time - the emerging dimension of plant virus studies. Journal of General Virology 91: 13–22. 19889925 10.1099/vir.0.015925-0

