# Supplementary figures and images for: The Recent Recombinant Evolution of a Major Crop Pathogen, Potato virus Y
Source: PLoS One. 2012 Nov 30;7(11):e50631. doi: 10.1371/journal.pone.0050631 (PMC3511492; doi:10.1371/journal.pone.0050631)

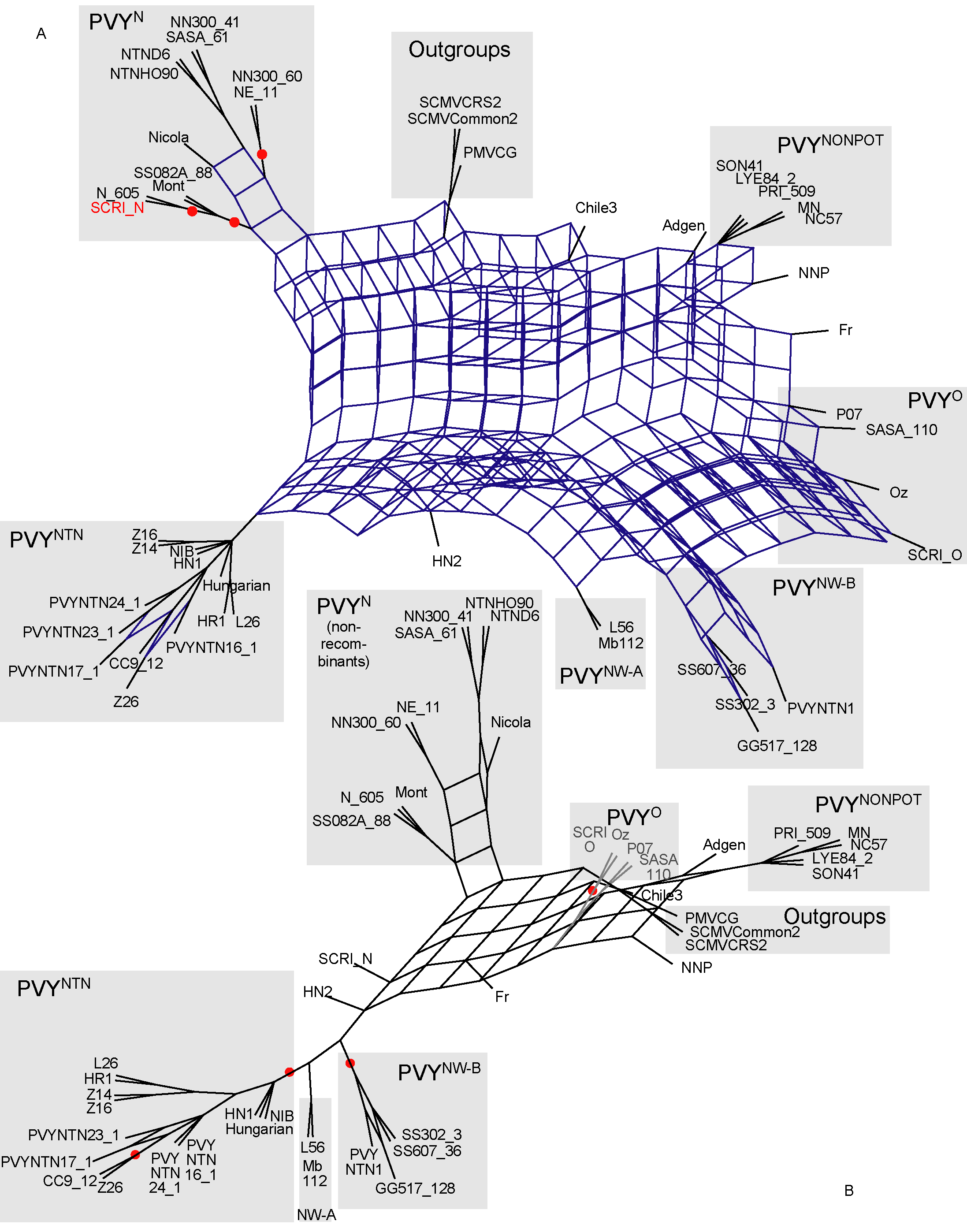

Supplement: Figure S1 — Phylogenetic networks summarised using SplitsTree a) from the 12 70% BS consensus trees in Fig. 3 ; b) from a single 70% BS consensus of the multi-labelled tree in Fig. 5 b. Nodes recovered in one network but contradicted in the other are indicated with red dots on the corresponding branches. Major PVY strains and recombinant clades are indicated. (TIF) [file pone.0050631.s001.tif]
