# Supplementary material for: The Recent Recombinant Evolution of a Major Crop Pathogen, Potato virus Y
Source: PLoS One. 2012 Nov 30;7(11):e50631. doi: 10.1371/journal.pone.0050631 (PMC3511492; doi:10.1371/journal.pone.0050631)
Supplement: Table S1 — Accessions details, including details regarding tip dates used in BEAST analyses and from who the information was obtained. (DOC) [file pone.0050631.s002.doc]

Table S1: Accessions details, including details regarding tip dates used in BEAST analyses and from who the information was obtained. Note the discrepancy between the date genome sequences were submitted to GenBank and the appropriate dates for use in molecular clock calibration; where the latter could not be confirmed the isolates in question were omitted from the molecular dating analyses. New isolates obtained for this study were assigned cryptic codes on collection to ensure that they could not subsequently be identified to a specific farm within South Africa.

| **Strain** | **Isolate** | **GenBank accession number** | **Country of origin** | **Genbank submission date** | **Accurate tip date confirmed with author** | **Notes regarding tip date** | **Source of information** |
| --- | --- | --- | --- | --- | --- | --- | --- |
| C | Adgen | AJ890348 | France | 2005 | 2003 | Adgen, ELISA company, use this as a positive control, necessarily maintained in culture. RNA isolated 2003 | Jörg Schubert (Julius Kühn-Institut, Institute for Biosafety of Genetically Modified Plants, Germany) |
| Chile | Chile3 | FJ214726 | Chile | 2008 | 2005 | RNA isolation in 2005 | Benoit Moury (INRA, France) |
| N | Mont | AY884983 | USA | 2005 | N/A |  |  |
| N | N605 | X97895 | Switzerland | 1996 | 1994 | RNA isolated 1994 | Pia Malnoë (Station Fédérale de Recherches en Production Végétale de Changins, Switzerland) |
| N | Nicola | AJ890346 | Germany | 2005 | 2003 | Isolated from potato in 2001, but since maintained in tobacco, RNA isolated 2003 | Jörg Schubert (as above) |
| N | NN300 41 | JN936422 | South Africa | 2011 | 2005 |  | This study |
| N | NTND6 | AB331515 | Japan | 2007 | N/A |  |  |
| N | NTNHO90 | AB331517 | Japan | 2007 | N/A |  |  |
| N | SASA-61 | AJ585198 | UK | 2003 | N/A |  |  |
| N | SS082A 88 | JN936433 | South Africa | 2011 | 2005 |  | This study |
| N/O | HN2 | GQ200836 | China | 2009 | N/A |  |  |
| N/O | SCRI-N | AJ585197 | UK | 2003 | N/A |  |  |
| NE | NE-11 | DQ157180 | USA | 2005 | N/A |  |  |
| NE | NN300 60 | JN936423 | South Africa | 2011 | 2005 |  | This study |
| NONPOT | LYE84.2 | AJ439545 | Spain | 2002 | 1984 | Isolated 1984, subsequently kept frozen, periodically maintained by means of re-infection in tobacco (brief; every c. 5 years); sequencing 1999 | Benoit Moury (as above) |
| NONPOT | MN | AF463399 | USA | 2001 | 1997 | RNA isolated 1997. | John P. Fellers/Steve Lommel (North Carolina State University, USA) |
| NONPOT | NC57 | DQ309028 | USA | 2005 | N/A |  |  |
| NONPOT | PRI-509 | EU563512 | Netherlands | 2011 | 2007 | RNA isolated in 2007 from a potato plant vegetatively propagated since 1938 | René van der Vlugt (Wageningen, The Netherlands) |
| NONPOT | SON41 | AJ439544 | France | 2002 | 1982 | Isolated 1982, used for reinfection of tobacco every approximately 5 years | Benoit Moury (as above) |
| NTN | CC9 12 | JN936416 | South Africa | 2011 | 2005 |  |  |
| NTN | HN1 | HQ631374 | China | 2009 | N/A |  |  |
| NTN | HR1 | FJ204166 | USA | 2008 | 2007 | RNA isolation 2007 | Alexander Karasev (University of Idaho, USA) |
| NTN | Hungarian | M95491 | Hungary | 1993 | 1992 | RNA isolated in 1992 | Vera Thole/Ervin Balazs (Institute for Plant Sciences, Hungary) |
| NTN | L26 | FJ204165 | USA | 2008 | 2008 | RNA isolation 2008 | Alexander Karasev (as above) |
| NTN | NIB | AJ585342 | Slovenia | 2003 | 2002 | RNA isolated from inoculated tobacco plants in 2002 | Nataša Barker (formerly Toplak; Scottish Crop Institute) |
| NTN | PVYNTN16 1 | JN936428 | South Africa | 2011 | 2007 |  | This study |
| NTN | PVYNTN17 1 | JN936429 | South Africa | 2011 | 2007 |  | This study |
| NTN | PVYNTN23 1 | JN936430 | South Africa | 2011 | 2007 |  | This study |
| NTN | PVYNTN24 1 | JN936431 | South Africa | 2011 | 2008 |  | This study |
| NTN | Z14 | JN936440 | South Africa | 2011 | 2009 |  | This study |
| NTN | Z16 | JN936441 | South Africa | 2011 | 2010 |  | This study |
| NTN | Z26 | JN936442 | South Africa | 2011 | 2005 |  | This study |
| NW-A | L56 | AY745492 | Canada | 2004 | N/A |  |  |
| NW-A | Mb112 | AY745491 | Canada | 2004 | N/A |  |  |
| NW-B | GG517 128 | JN936419 | South Africa | 2011 | 2005 |  | This study |
| NW-B | PVYNTN1 | JN936427 | South Africa | 2011 | 2007 |  | This study |
| NW-B | SS302 3 | JN936435 | South Africa | 2011 | 2010 |  | This study |
| NW-B | SS607 36 | JN936437 | South Africa | 2011 | 2010 |  | This study |
| O | Oz | EF026074 | USA | 2006 | N/A |  |  |
| O | P07 | U09509 | Canada | 1994 | N/A |  |  |
| O | SASA-110 | AJ585195 | UK | 2003 | N/A |  |  |
| O | SCRI-O | AJ585196 | UK | 2003 | N/A |  |  |
| O/N | Fr | X12456 | France | 1988 | 1986 | RNA isolation in 1986 | Chistophe Robaglia |
| O/NONPOT | NNP | AF237963 | Italy | 2000 | N/A |  |  |
| PMV | PMVCG | M96425 | USA | 1992 | 1989 | RNA isolated in 1989 | Vicki Vance (University of South Carolina) |
| SCMV | SCMVCommon | NC014038 | Argentina | 2009 | 2009 | Isolate made from sunflowers in 1994 and maintained in sunflowers until RNA was isolated in 2009. | Sergio Lenardon (INTA, Argentina) |
| SCMV | SCMVCRS | GU181200 | Argentina | 2009 | 2009 | Isolate made from sunflowers in 2005 and maintained in sunflowers until RNA was isolated in 2009. | Sergio Lenardon (as above) |
