# Supplementary material for: The Recent Recombinant Evolution of a Major Crop Pathogen, Potato virus Y
Source: PLoS One. 2012 Nov 30;7(11):e50631. doi: 10.1371/journal.pone.0050631 (PMC3511492; doi:10.1371/journal.pone.0050631)
Supplement: Table S2 — Summary of break points identified in the genomes of the isolates analysed in this study. (DOC) [file pone.0050631.s003.doc]

Table S2: Summary of break points identified in the genomes of the isolates analysed in this study. Breakpoint positions are according to the alignment with outgroups (before ‘/’) and relative to the sequence of isolate ‘Adgen’ (after ‘/’).

| Recombination # | Isolate/clade | Number of isolates | Begin breakpoint | End breakpoint |
| --- | --- | --- | --- | --- |
| 1 | NNP | 1 | 1/1 | 1287/592 |
| 2 | NE1 | 1 | 1/1 | 2707/2012 |
| 3 | NE2 | 1 | 1/1 | 2932/2237 |
| 4 | NTN/NW | 18 | 1/1 | 3107/2412 |
| 5 | NWB | 4 | 1186/491 | 3107/2412 |
| 6 | HN2 | 1 | 1/1 | 3209/2514 |
| 7 | PVYNTN1 | 1 | 3160/2465 | 6541/5847 |
| 8 | NTN | 13 | 6520/5825 | 9926/9180 |
| 9 | SCRI-N | 1 | 8568/7858 | 9053/8343 |
| 10 | Fr | 1 | 8623/7913 | 9052/8342 |
| 11 | HN2 | 1 | 6520/5825 | 9285/8575 |
| 12 | NNP | 1 | 9927/9181 | 10649/9674 |
| 13 | Fr | 1 | 10172/9426 | 10654/9679 |
